# Nano-Hybrid Au@LCCs Systems Displaying Anti-Inflammatory Activity

**DOI:** 10.3390/ma15103701

**Published:** 2022-05-22

**Authors:** Marcello Condorelli, Antonio Speciale, Francesco Cimino, Claudia Muscarà, Enza Fazio, Luisa D’Urso, Carmelo Corsaro, Giulia Neri, Angela Maria Mezzasalma, Giuseppe Compagnini, Fortunato Neri, Antonina Saija

**Affiliations:** 1 Department of Chemical Sciences, University of Catania, V.le A. Doria 6, 95125 Catania, Italy; marcello.condorelli@studium.unict.it (M.C.); ldurso@unict.it (L.D.); gcompagnini@unict.it (G.C.); 2 Department of Chemical, Biological, Pharmaceutical, and Environmental Sciences, University of Messina, Viale F. Stagno D’Alcontres 31, 98166 Messina, Italy; antonio.speciale@unime.it (A.S.); francesco.cimino@unime.it (F.C.); claudia.muscara@unime.it (C.M.); giulia.neri@unime.it (G.N.); 3 Department of Mathematical and Computational Sciences, Physical Sciences and Earth Sciences, University of Messina, Viale F. Stagno D’Alcontres 31, 98166 Messina, Italy; enza.fazio@unime.it (E.F.); carmelo.corsaro@unime.it (C.C.); angelamaria.mezzasalma@unime.it (A.M.M.)

**Keywords:** linear carbon chain, Au nanoparticles, cytotoxicity assay, anti-inflammatory effects, intestinal epithelial cells, TNF-α

## Abstract

Gold nanoparticles (Au NPs) have received great attention owing to their biocompatible nature, environmental, and widespread biomedical applications. Au NPs are known as capable to regulate inflammatory responses in several tissues and organs; interestingly, lower toxicity in conjunction with anti-inflammatory effects was reported to occur with Au NPs treatment. Several variables drive this benefit-risk balance, including Au NPs physicochemical properties such as their morphology, surface chemistry, and charge. In our research we prepared hybrid Au@LCC nanocolloids by the Pulsed Laser Ablation, which emerged as a suitable chemically clean technique to produce ligand-free or functionalized nanomaterials, with tight control on their properties (product purity, crystal structure selectivity, particle size distribution). Here, for the first time to our knowledge, we have investigated the bioproperties of Au@LCCs. When tested in vitro on intestinal epithelial cells exposed to TNF-α, Au@LCCs sample at the ratio of 2.6:1 showed a significantly reduced TNF gene expression and induced antioxidant heme oxygenase-1 gene expression better than the 1:1 dispersion. Although deeper investigations are needed, these findings indicate that the functionalization with LCCs allows a better interaction of Au NPs with targets involved in the cell redox status and inflammatory signaling.

## 1. Introduction

In the last decades, nanotechnological strategies combined with a supramolecular approach allowed us to reach geometrically well-defined and responsive nanodevices [1,2] with a relevant impact in several fields [3,4,5]. In particular, the engineering of suitable nanomaterials provides high-performance nanomedicine systems including drug/gene delivery carriers, imaging tools, tissue scaffolds, and biosensors [6,7,8,9]. For this purpose, carbon-based nanomaterials are described in depth in scientific literature and, since containing different functional groups, can easily interact with several biomolecules [10,11,12,13,14]. For these reasons carbon-based materials including hybrid metallic nanoparticles-organic nanostructure are being quite used to design new biomedical applications taking advantage of their therapeutical activity, or as drug delivery vehicles [15,16].

Given the remarkable technological breakthroughs offered by these carbon allotropes and the more recently nanomedicine research attention on natural polyynes isolated by fungi, bacteria, plants, the interest in synthetic linear carbon chains (i.e., polyynes/carbyne, the one-dimensional allotrope of carbon composed of sp-hybridized carbon atoms), and their unusual potential properties is heavily increased [17,18,19]. Natural polyynes are characterized by a great variety of structural features (oxygen-containing functional groups, ring chain, aromatic rings, etc.) which are suitable in view of green biological applications such as antimicrobial, antibacterial, antitumoral, and pesticidal activities [19,20]. These properties open the way to design chemical artificial polyynes able to (i) interact with proteins; (ii) behave as pro-drug systems and (iii) join DNA-complexing substructures. Moreover, relevant clinical results have reached conjugating natural enediynes with monoclonal antibodies [21], while Del Rosso et al. demonstrated the high biocompatibility of a carbenoid-gold (Au@Carbynoid) nanocomposite stabilized by using Pluronic F127 as well as its potentiality in photodynamic therapy and as drug delivery system [22]. These scientific results highlight the potentiality of hybrid linear carbon chains-gold (Au@LCC) nanocomposites in view of biomedicine applications.

In the last ten years, the synthesis of gold nanoparticles (Au NPs) and linear carbon chains (LCCs) with finite length was successfully carried out by a green approach: the Pulsed Laser Ablation in Liquid (PLAL) technique. In fact, the PLAL approach avoids the use of organic solvents, purification steps, or heating treatments [23], in contrast to the common chemical strategies employed to reach natural polyynes and their derivatives. Spectroscopic analyses confirm that the achieved LCCs are hydrogen capped sp hybridized carbon structures with alternating single and triple bonds [24,25], while Au NPs are spherical with an average size of 5 nm [25,26]. However, the poor colloidal stability of the obtained LCCs leads to reorganization phenomena via crosslinking reaction in sp${}^{2}$ structures [27]. To overcome this limit, the employment of spacers at the end of an sp-carbon chain, such as the embedding of these carbon chains in solid matrices (PVA, SiO${}_{2}$, metal nanoparticles), is an efficient strategy that helps to avoid sample degradation [28,29,30,31,32,33]. As reported by Pan et al. [34], the carbon-hydrogen bonds in carbyne can be cleaved with the help of gold catalysts under the favorable thermodynamic environment provided by PLAL and because the unique configuration of two carbon atoms in an alcohol molecule matches the elementary entity of carbyne. Catalyst properties of Au NPs have also been exploited in several reactions producing hybrid innovative systems [35,36,37,38]

It is further important to consider that Au-based compounds have a long history of being used for therapeutic purposes. In recent times, Au NPs have shown great potential in the areas of imaging, diagnosis, therapy, and drug delivery owing to their exceptional physicochemical properties [39]. Au nanosystems have been utilized as effective therapeutic agents for the treatment of some inflammatory diseases such as rheumatoid arthritis [40,41]. Further, lower toxicity in conjunction with anti-inflammatory effects was reported to occur with Au NPs treatment [42,43]. However, it is well known that cell interaction and uptake, as well as the bioproperties and toxicity of Au NPs, depend on several factors including size, physicochemical stability, morphology and aggregation state, coating, and functionalization [44,45].

Herein, we prepared a hybrid nanosystem (Au@LCCs) based on the growth of carbon linear chains within an Au nanocolloidal solution, carrying out a pulsed laser ablation process. First, we verified, as well known from literature data, that also in our case, AuNPs improved the colloidal stability of polyynes. Then, the morphological-structural properties were investigated. However, the main focus of our research is to study the potential therapeutic effectiveness of Au@LCCs as an anti-inflammatory agent at the intestinal level, an aspect, up to today, largely unexplored, unlike other Au NPs biological effects. For this aim, we used an in vitro model of intestinal inflammation induced on differentiated colon Caco-2 cells by exposure to tumor necrosis factor-α (TNF-α); the Caco-2 cell monolayer model was chosen because it well mimics the colon epithelial cell layer, both morphologically and functionally [43]. Importantly, the findings obtained by using three Au@LCCs preparations with different Au/LCCs ratios allow us to highlight that the biological properties of the Au NPs can be modulated by the functionalization with LCCs.

## 2. Materials and Methods

### 2.1. Au@LCC Synthesis and Characterization

**Figure 1:**
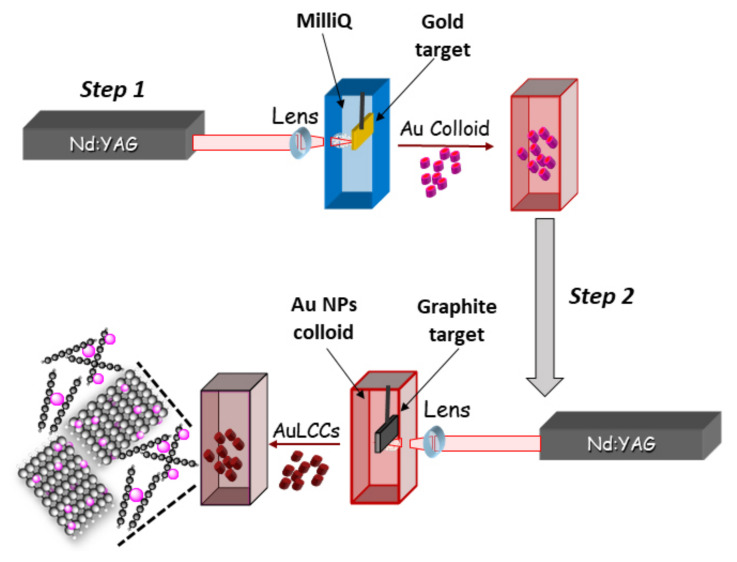
Steps followed during PLAL processes and representative scheme of the so synthesized Au@LCCs hybrid system.

Surfactant-free Au NPs and LCCs were separately prepared by ablating respectively Au and graphite (99.9% purity) targets (purchased from GoodFellow, Hamburg, Germany) in Millipore grade water (10 mL) to which 1 mM of NaCl at pH 9 has been added. Briefly, a plasma created by the impact of a pulsed laser beam onto the target was generated and confined in the surrounding liquid. The steps followed during PLAL processes are shown in Figure 1 together with a representative scheme of the so synthesized Au@LCCs hybrid system.

The optimization of the PLAL procedure (acting on ablation time, laser fluence, and target-substrate distance) was made to reach a good compromise for observing appreciable spectroscopic signals and simultaneously preventing LCCs degradation. The incident beam is the 1064 nm line of the Continuum, Surelite II model Nd:YAG laser (pulse duration = 5 ns, repetition rate = 10 Hz). The laser ablation is performed for 20 min with a fluence at the target surface of about 0.98 J/cm${}^{2}$, high enough to overcome the graphite and Au ablation threshold. Then, we prepared three different Au@LCCs dispersion with different Au:LCCs ratios. In these cases, the polycrystalline graphite target was ablated into the Au colloidal dispersion (10 mL) and used as the medium. To modulate the concentration of LCCs, we varied the ablation time up to 20 min. The concentration of the synthesized samples was estimated by weighing the metal and graphite targets, before and after the ablation process. Before the weighing, both targets were rinsed sonicated, and dried with N${}_{2}$. The calculated concentrations of the three different Au@LCCs dispersions are: (I) 373 μg Au/mL and 360 μg LCCs/mL (hereafter indicated as Au@LCCs 1:1); (II) 373 μg Au/mL and 145 μg LCCs/mL (Au@LCCs 2.6:1); (III) 128 μg Au/mL and 365 μg LCCs/mL (Au@LCCs 1:2.9).

**Figure 2:**
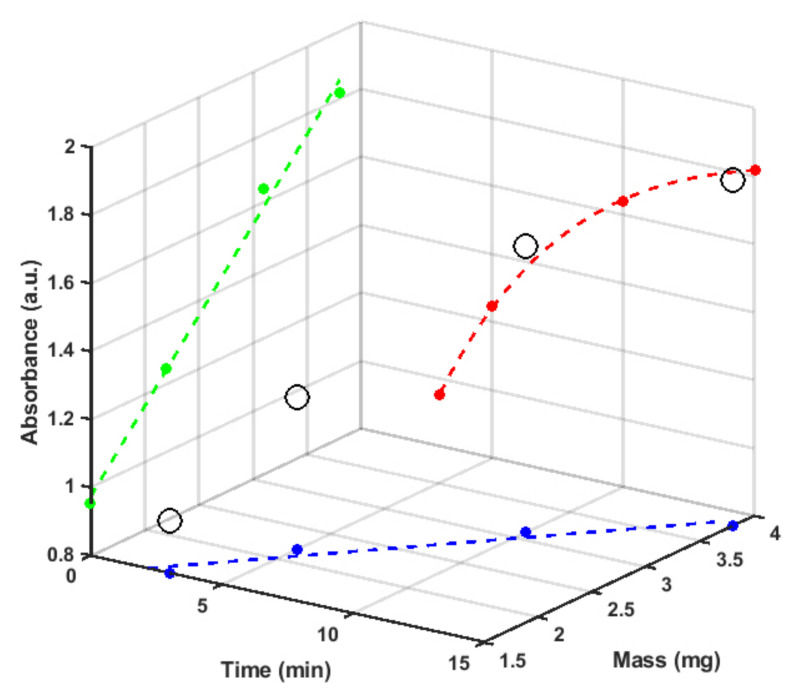
3D plot of the optical absorbance value of the 226 nm peak as a function of ablation time (red data projection) and the amount, expressed in milligrams, of the ablated graphite target (green data projection) in a water volume of 10 mL (which is proportional to the amount of LCC content in the colloidal solution).

UV–vis optical transmission and Raman measurements were performed to test the presence of LCCs through their characteristic absorption (200–500 nm) and vibrational features (1700–2100 cm${}^{-1}$) [29]. Figure 2 reports a three-dimensional plot of the LCC optical absorbance values at 226 nm as a function of ablation time (red data projection) and the amount (Mass) of target ablated (green data projection) expressed in milligrams. As expected, the blue and green data projections show a linear relationship between mass and ablation time as well as between the LCCs optical characteristics and Mass.

UV–vis optical transmission measurements, carried out using an Agilent Cary V60, were measured in the 190–600 nm range immediately after the ablation process and after a month to monitor the stability of the samples. Raman spectra were performed at room temperature on freeze-dried samples by a WITec alpha 300 confocal Raman apparatus exciting with a 532 nm laser line of a Coherent Compass Sapphire Laser. An integration time of 5 s, with an accumulation number of 8 was used for the Raman analyses. Using the Horiba NanoParticle Analyzer SZ-100, the Zeta potential was quantified using a laser Doppler method based on the principle of electrophoretic mobility under an electric field. To investigate sample morphology, the colloidal solutions, deposited on platinum grids, were dried in air for 5 h. Scanning Transmission Electron Microscopy (STEM) analyses were carried out using a Zeiss-Gemini 2 electron microscope operating at 30 kV. The surface chemical composition was investigated by deconvolving XPS survey and high-resolution profiles with Gauss–Lorentzian shape functions with the same Full Width at Half Maximum, FWHM (about 1.4 eV), for all of the considered subbands. XPS spectra were acquired using a Thermo Scientific system, equipped with a monochromatic Al-Kα source (1486.6 eV), operating in a constant analyzer energy (CAE) mode with a pass energy of 20 eV for high-resolution spectra and a spot size of 400 μm. Transmission Electron Microscopy (TEM) images were collected by a JEOL JEM 2010 transmission electron microscope operating at an acceleration voltage of 200 kV. X-ray diffraction (XRD) patterns were recorded in the 2 theta range from 20° to 80° using a Bruker D8 Advance X-ray diffractometer with Cu Kα radiation (1.5406 Å).

### 2.2. Cell Cultures

The fibroblast NIH/3T3 cell line was obtained from the American Type Culture Collection (ATCC, Rockville, MD, USA). Cells were cultured in Dulbecco’s modified essential medium (DMEM) supplemented with 10% fetal bovine serum (FBS), 4 mM L-glutamine, streptomycin, and penicillin, maintained in an incubator with a humidified atmosphere containing 5% CO${}_{2}$ at 37 °C.

Caco-2 intestinal epithelial cells, obtained from ATCC, were grown in DMEM supplemented with 10% FBS, 4 mM L-glutamine, 1% non-essential amino acids, 100 U/mL penicillin, and 100 μg/mL streptomycin. Cells were maintained at 37 °C in a humidified atmosphere with 95% air and 5% CO${}_{2}$.

### 2.3. Citotoxicity Assay

The biocompatibility on NIH/3T3 cells of Au NPs, LCCs, and the three different Au@LCCs dispersions (Au@LCCs 1:1, Au@LCCs 2.6:1, Au@LCCs 1:2.9) were investigated using sulforhodamine B (SRB; a dye binding to cellular proteins) assay, as previously described [46] with some modifications. Briefly, NIH/3T3 cells (3.5 × 10${}^{4}$ cells/well) were plated in 96-wells cell plates and, after 24 h, semi-confluent monolayers were treated for 24 or 48 h with different concentrations of: AuNPs (range: 4–32 μg/mL); LCCs (range: 11.8–94.2 μg/mL); or the three different Au@LCCs dispersions (range: 4–32 μg/mL expressed as Au; Au:LCCs ratios: 1:1, 2.6:1, 1:2.9). Control cells were exposed to the same volumes of the vehicle alone (1mM NaCl). Then, cells were fixed using 10% trichloroacetic acid for 1 h at 4 C. After fixation, cells were washed twice with water and incubated with SRB (0.4% *w*/*v* in 1% acetic acid) for 30 min at RT, followed by four washes with 1% acetic acid. The bound dye was solubilized in 1 mL of 10 mM Tris base solution and the absorbance was measured at 565 nm. Cell viability results are expressed as the percentage of viable cells in treated samples with respect to control cells.

### 2.4. Intestinal Epithelial Cells Inflammation Model

Caco-2 cell monolayers were prepared by seeding cells at 4 × 10${}^{4}$ per cm${}^{2}$ on the upper side of transwell inserts (0.4 μm pore size; Greiner Bio-One Italia S.r.l., Cassina de’ Pecchi, Milano, Italy) and cultured for 18 days post confluence to obtain fully differentiated cells [47]. Monolayer integrity and formation of tight junctions (TJ) were assessed by measurement of Trans-Epithelial Electrical Resistance (TEER) by using a Millicell-ERS Voltohmmeter (Millipore, MA, USA). Monolayers used in this study had TEER values ≥600 Ω·cm${}^{2}$. Differentiated Caco-2 monolayers, prepared as above described, were pretreated or not with different concentrations of Au NPs (range: 16–32 mg/mL), LCCs (range: 6.2–30.9 mg/mL) or of two Au@LCCs dispersions (Au:LCCs ratios: 1:1 and 2.6:1; range: 16–32 mg/mL expressed as Au) for 24 h added only to the apical compartment of the transwell inserts. Used preparations were always freshly prepared. After 24 h, cells were washed twice with Dulbecco’s phosphate-buffered solution (DPBS) and then exposed for 6 h to 50 ng/mL TNF-α added in both the apical and the basolateral compartments of the transwell inserts. TNF-α concentration was chosen on the basis of preliminary experiments indicating that exposure to 50 ng/mL significantly decreased TEER value already after 3 h compared to the untreated control cells [48].

### 2.5. Real-Time Pcr

RNA was extracted using the E.Z.N.A. Total RNA Kit I following manufacturer’s instructions (OMEGA Bio-Tek VWR), quantified with Quant-iT^TM^ RNA assay kit by QUBIT fluorometer (Invitrogen, Milan, Italy), and reverse transcripted with the M-MLV reverse transcriptase. 7300 Real-Time PCR System (Applied Biosystems, Monza, Italy) coupled with the Sybr green JumpStart^TM^ Taq Ready Mix kit was used for the assessment of gene expression. The specific primers used were: 18S rRNA, forward, 5′-GTA ACC CGT TGA ACC CCA TT-3′, reverse, 5′-CCA TCC AAT CGG TAG TAG CG-3′; TNF, forward, 5′-CCA GGC AGT CAG ATC ATC TTC TC-3′, reverse, 5′-AGC TGG TTA TCT CTC AGC TCC AC-3′ [49]; heme oxygenase-1, forward, 5′-CAA CAT CCA GCT CTT TGA GG-3′, reverse, 5′-AGA AAG CTG AGT GTA AGG AC-3′ [50]. The fold increase of mRNA expression, compared with the control cells not pretreated and not exposed to TNF-α, and corrected with 18S rRNA housekeeping gene, was determined using the 2${}^{-\Delta \Delta Ct}$ method [51].

### 2.6. Statistical Analysis

All the experiments were performed in triplicate and repeated three times. Results are expressed as mean ± SD from 3 experiments and statistically analyzed by a one-way or a two-way ANOVA test, followed by Tukey’s HSD, using the statistical software ezANOVA [52]. Differences in groups and treatments were considered significant for *p*< 0.05.

## 3. Results

### 3.1. Physico-Chemical Characterization of Au@LCC Nanocolloids

LCCs, due to their intrinsic chemical nature, are extremely unstable and very reactive. Depending on the chain length, a cross-linking reaction usually happens towards the more stable sp${}^{2}$ phase. The longer the chain, the more unstable is the system. Apart from the chain length, the functionalization of the end group has a crucial role in characterizing both corresponding physicochemical properties and stability. For instance, it has been recently proven that p-conjugated, sp${}^{2}$ hybridized, end groups from one side can enhance the conjugation of the system and, from the other side, can increase the system stability [53,54]. Although it is a matter of debate, it seems that these end groups can determine the bond length alternation (BLA) and chain length of the sp-backbone with the aim to obtain cumulene-like systems with enhanced metallic character (e.g., a zero band-gap) [53,54]. In this work, we first analyze the physicochemical properties of our Au@LCC nanocolloids prepared by the green PLAL technique. Then, we also investigate their toxicity and anti-inflammatory action, knowing that both carbon-based nanomaterials and Au NPs are interesting materials for biomedical applications [55,56].

**Figure 3:**
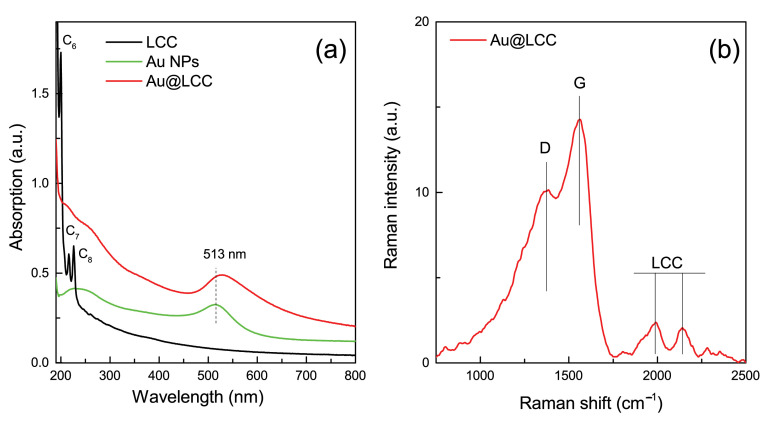
(**a**) UV-Vis absorbance spectra of LCC (black line), Au NPs (green line) and Au@LCC (1.1 ratio) (red line); (**b**) Raman spectra of Au@LCC.

Au@LCC optical absorption fingerprints of the electronic transitions of LCCs (assigned mainly to C${}_{6}$H${}_{2}$, C${}_{7}$H${}_{2}$ and C${}_{8}$H${}_{2}$ species) [24] and of the surface plasmon resonance (SPR) of Au NPs [57] are observed between 190 and 250 nm and in the 510–550 nm range, respectively (see Figure 3a). The UV-Vis absorbance spectra of Au and LCC pristine samples are also shown for comparison. In presence of LCCs, a shift of Au SPR peak towards the higher wavelength has been observed, whereas the characteristics of LCC features are not well resolved in the Au@LCC sample.

The Raman spectrum of the Au@LCC sample (Figure 3b) shows the typical vibrational modes due to the amorphous sp${}^{2}$ carbon species resulting from the overlap of the so-called G band (centered at about 1574 cm${}^{-1}$) and the D band (centered at about 1373 cm${}^{-1}$) [58] and the vibrational modes at around 1984 cm${}^{-1}$ and 2142 cm${}^{-1}$), ascribed to LCCs containing 6–8 carbon atoms (triple bonds (C≡C) of carbon) [59,60]. LCC contributions are not evident in the bare LCC because of their instability in absence of end groups that hinders their degradation, but their features are visible in the Au@LCC sample. This behavior has been assumed to be due to the contribution of the bonds connecting the chain with the metal NPs [61,62] which, at the same time, stabilizes and enhances the Raman signal. The interaction between parallel chains is expected to be weaker than the interactions between neighboring carbon atoms in a chain. We conclude that the Van der Waals forces are responsible for the formation of bundles composed by sp and sp${}^{2}$ hybridized carbons [34] interconnecting Au NPs.

Simultaneously, the observed SEM/TEM and Raman features are in good agreement with computational electron-density profiles [63,64] indicate that Au atoms have significant interaction with the LCCs, which leads to the electronic stabilization of LCCs, while intercalated Au atoms or clusters lead to LCCs kinking that results from the interaction of the electronic densities of the carbon chains and Au NPs [65].

**Figure 4:**
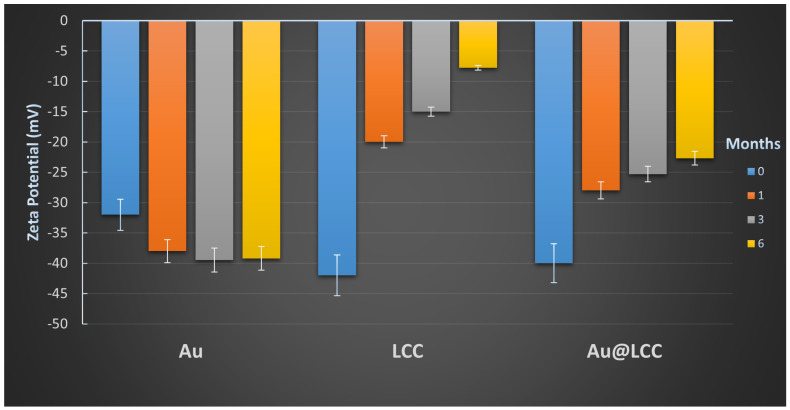
Zeta potential values measured for the three considered samples.

The Zeta potential value, which measures the electrostatic potential that exists on the Au NPs and LCCs surface, reveals information regarding the surface charge and stability of the nanoparticulate formulation (Figure 4).

Immediately after the ablation process, Au NPs, LCCs, and Au@LCC systems show a negative charge of −32 mV, −42 mV, and −40 mV, respectively. These data indicated the relative stability of the colloidal solution. After one month of storage, Zeta potential values are −38 mV for Au NPs, −20 mV for LCCs, and −28 mV for Au@LCC samples. After three-six months, LCCs alone have substantially changed into sp${}^{2}$, carbon-based materials, while the surface charge of Au@LCC samples is slightly increased. These changes rule out the possibility of aggregation processes, while a partial shielding of the Au NPs surface charges could occur. Thus, the LCC arrangements formed on the surface reduce the electrophoretic mobility, so stabilizing the system for its relatively long-term storage.

**Figure 5:**
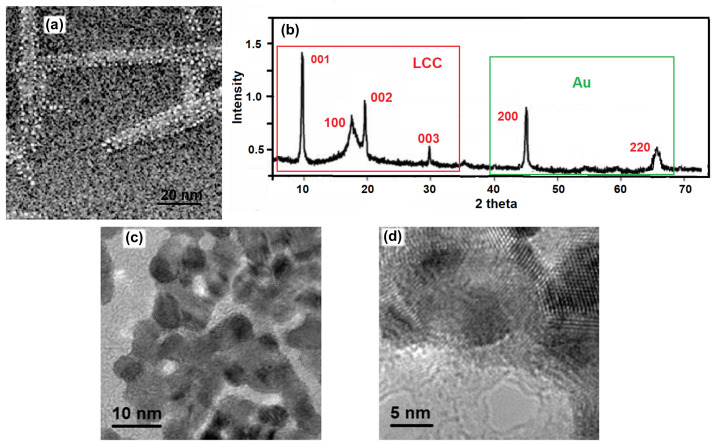
SEM image (**a**), corresponding XRD spectrum (**b**) and STEM images acquired in different points of Au@LCCs sample (**c**,**d**) with 1:2.9 ratio.

SEM image of the Au@LCCs sample with 1:2.9 ratio shows well defined rod-like structures (Figure 5a). The corresponding XRD pattern (Figure 5b) proposes the typical structure of LCCs [34]. The XRD spectrum of Au NPs, which decorate the LCCs surface, shows two peaks at about 43.6° and 64.3° assigned to the (200) and (220) planes of face-centered cubic (fcc) structure of Au NPs (JCPDS card no. 04-0784) (right inset of Figure 5b). Further, the size of Au NPs estimated using the well-known Scherrer formula is in good agreement with that observed by microscopic techniques. The STEM image of the Au@LCCs sample (Figure 5c) displays polyynes-based bridges connecting the neighboring Au NPs, whose average size is about 8 nm. Moreover, the high magnification STEM image in Figure 5d, acquired at a different point to that of Figure 5c, shows filaments that form a random array connecting metal islands.

**Figure 6:**
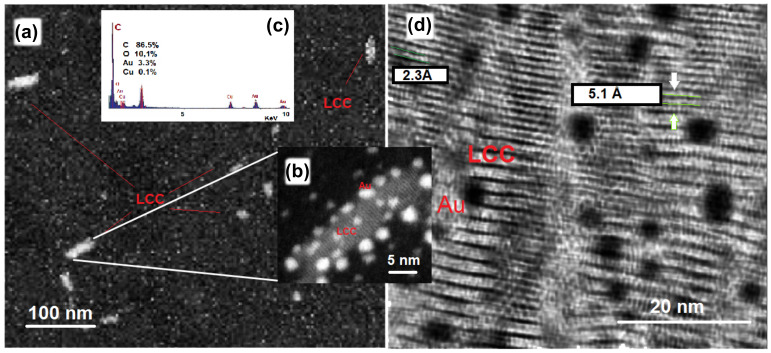
SEM-EDX data (**a**–**c**) and HR-TEM image (**d**).

To further investigate the distribution of Au NPs and LCCs and their size, we analyze in detail the morphological properties of the Au@LCCs sample with the high Au content (2.6:1 ratio). Figure 6 shows SEM-EDX data (a–c) and HR-TEM image (d). Randomly distributed chains are evident from the SEM image. The corresponding EDX spectrum indicates that the sample is composed mainly of C and O, Au, Cu (this latter coming from the grid used to carry out TEM analysis). HR-TEM image (d) shows both the formation of bundles of carbons (C-bundles) having a rod-like shape (8 to 20 nm in width and 20 to 80 nm in length) decorated with nearly spherical Au NPs (SEM Figure 6b). In some regions, field-aligned polarized bundles end-capped with Au NPs prevail. The distances between the linear carbon structures range between about 2–5 Å.

**Figure 7:**
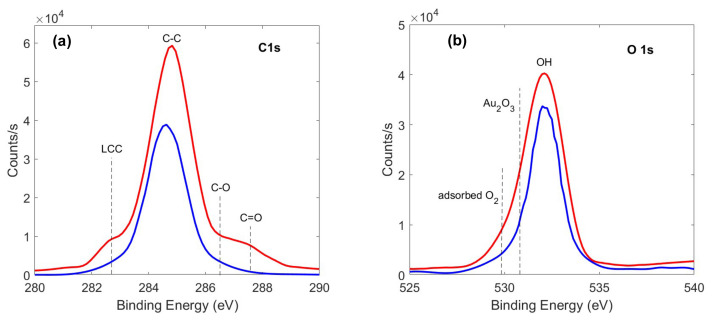
C 1s (**a**) and O 1s (**b**) XPS profiles of Au (blue line) and Au@LCCs (red line) nanosystems.

Finally, the surface chemical bonding configurations were determined by analyzing XPS profiles of Au NPs and Au@LCCs samples. We outline that no significant information was obtained by the XPS profiles of LCCs since, after the deposition and exposure to air (solid sample), the non-stabilized LCCs change in amorphous carbon. Thus, XPS analyses were carried out only on Au and Au@LCCs samples providing C, O, and Au percentages respectively equal to 63.8, 33.7, and 2.5 for Au NPs, and 72.0, 26.1, and 1.9 for Au@LCCs. No substantial differences have emerged comparing O 1s and C 1s lineshapes of Au and Au@LCCs samples (Figure 7). The O 1s main peak at 531.8 eV is ascribed to [OH]- related species, so excluding the oxidation of Au NPs or Au NPs@LCC. Further, the features ascribed to C–O and C=O bonds, are not very pronounced confirming previous results reported in ref. [33].

**Figure 8:**
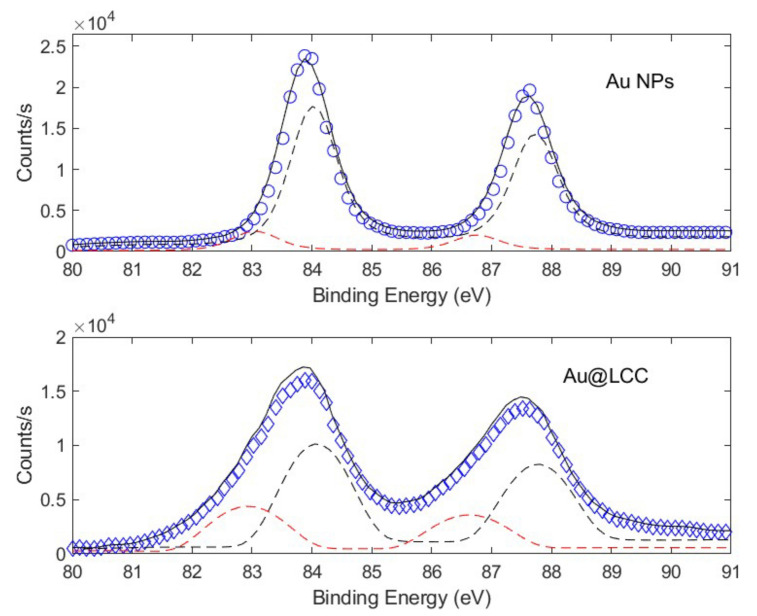
Au 4f lineshape deconvolution for NPs (**top**) and AuNPs@LCC (**bottom**) samples.

Otherwise, the formation of Au-C bonds has been evidenced by comparing the Au 4f spectra of Au and Au@LCCs samples, respectively (see Figure 8). Au@LCCs sample maintains the energy position of the main peaks referred to as the position of the metallic gold reference but shows a noticeable broadening in the lower energy region of the Au 4f spectrum. This low energy contribution in the Au@LCCs was due to the formation of Au–C bonds [66,67].

To quantify the amount of Au-LCC bonds, the Au 4f core-level photoemission peaks were deconvoluted with Gauss–Lorentzian shape functions with the same FWHM (about 1.4) for all of the considered subbands. Specifically, Au 4f profiles were reproduced by considering two contributions: the doublet ascribed to the Au 4f${}_{7/2}$ and 4f${}_{5/2}$ signals in the gold metal, centered at 83.9 and 87.5 eV, and to the 4f doublet, attributed to the Au-C bonds, at the binding energies of about 82.9 and 86.5 eV, respectively. The fitting results are shown in Figure 8. In the presence of LCCs, the percentage of the Au-C bonds (39.8%) significantly increases with respect to the Au NPs one (3.6%), while the amount of the Au metallic contribution decreases (from 96.4 down to 60.2%).

### 3.2. Biocompatibility on NIH/3T3 Fibroblasts

**Figure 9:**
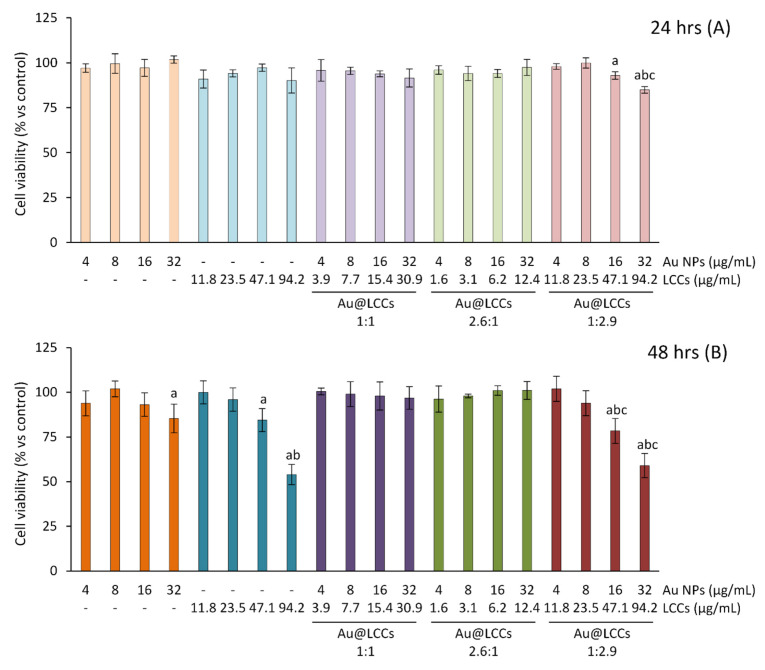
**NIH/3T3 cell viability.** NIH/3T3 fibroblasts were exposed to Au NPs (range: 4–32 μg/mL), LCCs (range: 11.8–94.2 μg/mL) or Au@LCCs dispersions (range: 4–32 μg/mL expressed as Au) for 24 h (**A**) or 48 h (**B**). Cell viability was then evaluated by the SRB assay. Results (reported as mean ± SD of three experiments) are expressed as percentage of cell viability in treated samples with respect to control cells. ${}^{a}$ *p*< 0.05 vs. CTR; ${}^{b}$ *p*< 0.05 vs. lower concentrations of the same treatment; ${}^{c}$ *p*< 0.05 vs. same concentration (expressed as μg/mL of Au) of the other Au@LCCs.

NIH/3T3 fibroblasts were used to test the biocompatibility of different concentrations of Au NPs, LCCs, and of the three different Au@LCCs dispersions (Au@LCCs 1:1, with Au 373 mg/mL and LCCs 360 mg/mL; Au@LCCs 2.6:1, with Au 373 mg/mL and LCCs 145 mg/mL; Au@LCCs 1:2.9, with Au 128 mg/mL and LCCs 365 mg/mL). The Au NP tested concentrations were in the range 4-32 μg/mL, while LCC cytotoxicity was evaluated at concentrations up to 94.2 μg/mL. No significant change in cell viability was observed following 24 h exposure to Au NPs or LCCs. As measured after 48 h exposure, LCCs at 47.1 and especially at 94.2 μg/mL appeared to be significantly cytotoxic, in a dose-dependent way; furthermore, a slight (although statistically significant) decrease in cell viability was found at the higher tested Au NPs dose (32 μg/mL). We could not verify the cytotoxicity of higher Au NP concentrations (>32 μg/mL) since they required a medium dilution which was incompatible with cell survival for nutrient deprivation. Thus, for this reason, Au@LCCs were tested at concentrations each up to Au 32 μg/mL. As to the Au@LCCs, no evidence of cytotoxicity was observed when cells were exposed for 24 or 48 h to Au@LCCs in the ratio 1:1 (32/30.9 μg/mL) and 2.6:1 (32/12.4 μg/mL). Conversely, as presumable on the basis of the results reported above, the Au@LCCs 1:2.9 dispersion appeared to be cytotoxic at the two higher tested concentrations (16/47.1 and 32/94.2 μg/mL), both at 24 and 48 h exposure (Figure 9). Thus, given the significant cytotoxicity of Au@LCCs 1:2.9, this preparation was excluded from the following experiments in Caco-2 cells.

### 3.3. Anti-Inflammatory and Antioxidant Effects on Intestinal Epithelial Cells Treated with Tnf-α

The anti-inflammatory properties of Au NPs have received increasing interest for their capability to regulate inflammatory responses in several tissues and organs [68]. In fact, Au NPs were shown to alleviate lipopolysaccharide (LPS)-induced inflammatory response by inactivation of the nuclear factor kappa-B (NF-κB) and Janus kinase 2 (JAK2)/signal transducer and activator of transcription 3 (STAT3) signaling pathways in RAW 264.7 macrophages [69]. Additionally, Au NPs could reduce high glucose-induced inflammation in macrophages via the tuberin-mammalian target of rapamycin (mTOR)/NF-κB pathway [70]. However, Sumbayev et al. [71], using human myeloid leukemia THP-1 cells exposed to the interleukin (IL) IL-1β, hypothesized that the anti-inflammatory activity of Au NPs may be attributed mainly to their extracellular interactions with IL-1β which aggregates around Au NPs, thus inhibiting IL-1β binding to cellular receptors. However, little is known about the possible application of Au NPs in the treatment of intestinal inflammation. Au NPs were shown able to inactivate the NF-κB and ERK/JNK pathways protecting against LPS-induced damage in colonic epithelial NCM460 cells [72]. Furthermore, Au NPs reversed the effect of LPS treatment on TJ proteins including ZO-1 and occludin, and proinflammatory cytokines, including IL-6, TNF-α, inducible nitric oxide synthase, and cyclooxygenase 2. The LPS-stimulated decrease in transepithelial permeability was rescued by Au NPs; moreover, Au NPs can upregulate B-cell lymphoma 2 (Bcl-2) and downregulate Bax and C-caspase-3 to reverse the LPS-stimulated apoptosis in this experimental model. Abdelmegid et al. [73] reported the protective effect of Au NPs on BABL/c adult mice treated with sodium sulfate (DSS) to induce ulcerative colitis. The findings indicated an overall improvement of DSS-induced adverse effects following Au NPs treatment, with decreased malondialdehyde levels in colon homogenates, an about normal colon structure and a significantly decreased collagen fibers content. These effects might be attributed to the antioxidant and the anti-inflammatory properties of Au NPs; the authors hypothesized that Au NPs could ameliorate the progression of inflammation by decreasing the pro-inflammatory cytokine IL-17 expression at the colon level.

**Figure 10:**
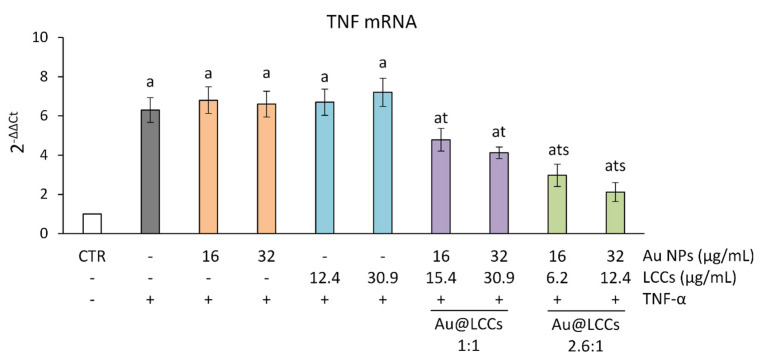
**TNF gene expression in differentiated Caco-2 cells.** The Caco-2 monolayers were pretreated or not with Au NPs (range: 16–32 μg/mL), LCCs (range: 6.2–30.9 μg/mL) or the Au@LCCs dispersions (range: 16–32 μg/mL expressed as Au) for 24 h, and subsequently exposed to 50 ng/mL TNF-α for 6 h. Cultures treated with the vehicles alone were used as controls (CTR). Results (reported as mean ± SD of three experiments) are expressed as 2${}^{-\Delta \Delta Ct}$. 18S rRNA was used as housekeeping gene. ${}^{a}$ *p*< 0.05 vs. CTR; ${}^{t}$ *p*< 0.05 vs. TNF-α; ${}^{s}$ *p*< 0.05 vs. Au@LCCs 1:1 treatments + TNF-α.

So, with the aim to demonstrate if Au@LCCs can induce an anti-inflammatory effect and to verify how the activity of Au NPs is modulated by functionalization with LCCs dispersions, Au@LCCs, in the ratio 1:1 and 2.6:1 and at concentrations each up to 32 μg/mL Au, were studied in an in vitro experimental model of intestinal inflammation consisting of intestinal epithelial Caco-2 cells exposed to TNF-α. Cytokines and chemokines are critical for intestinal epithelial homeostasis and responses to disease. TNF-α can increase the expression of proinflammatory cytokines, chemokines, adhesion molecules, and other inflammatory mediators; in particular, this cytokine induces the transcription of proinflammatory genes such as TNF, IL6 and IL8 [48,49], mainly through the activation of NF-κB pathway, leading to inflammation and tissue damage. None of the treatments per se affected the basal levels of TNF gene expression, demonstrating that neither Au NPs (16–32 μg/mL), nor LCCs (12.4–32.9 μg/mL) or the two tested Au@LCCs dispersions induce a pro-inflammatory response in Caco-2 cells (data not shown). TNF-α exposure, instead, induced a significant increase in TNF gene expression compared to control (Figure 10). While Au NPs and LCCs did not show any anti-inflammatory activity, TNF overexpression induced by TNF-α exposure was dose-dependently inhibited by the pretreatment with both the Au@LCCs dispersions tested. Moreover, the Au@LCCs 2.6:1 showed a significantly higher anti-inflammatory activity than the 1:1 dispersion (Figure 10). Although not well characterized and understood at the cellular and molecular level, the biological activity of Au NPs has been reported by several authors [74], conversely, there is no evidence of potential bioproperties of inorganic graphene-derived LCCs [75]. So the observed anti-inflammatory effects need to be ascribed to Au NPs contained in the hybrid Au@LCC colloid.

**Figure 11:**
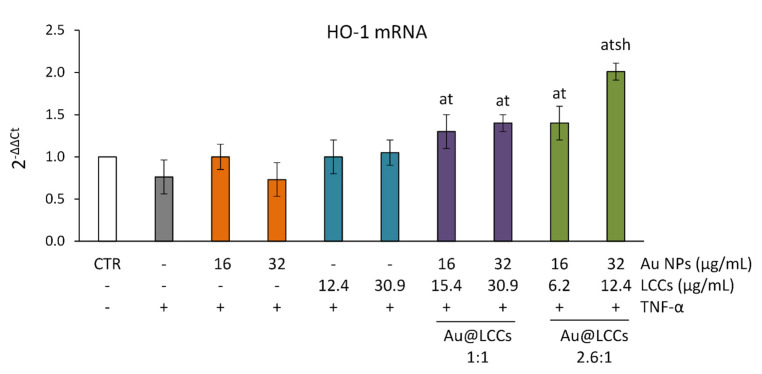
**HO-1 gene expression in differentiated Caco-2 cells.** The Caco-2 monolayers were pretreated or not with Au NPs (range: 16–32 μg/mL), LCCs (range: 6.2–30.9 μg/mL) or the Au@LCCs dispersions (range: 16–32 μg/mL expressed as Au) for 24 h, and subsequently exposed to 50 ng/mL TNF-α for 6 h. Cultures treated with the vehicles alone were used as controls (CTR). Results (reported as mean ± SD of three experiments) are expressed as 2${}^{-\Delta \Delta Ct}$. 18S rRNA was used as housekeeping gene. ${}^{a}$ *p*< 0.05 vs. CTR; ${}^{t}$ *p*< 0.05 vs. TNF-α; ${}^{s}$ *p*< 0.05 vs. Au@LCCs 1:1 treatments + TNF-α; ${}^{h}$ *p*< 0.05 vs. lower dose of Au@LCCs 2.6:1 + TNF-α.

Furthermore, the potential mechanisms underlying the anti-inflammatory and antioxidant effects of Au@LCCs were investigated also by evaluating their capability to modulate heme oxygenase-1 (HO-1) gene expression. HO-1 is an enzyme specialized in degrading heme and is assembled with biliverdin, carbon monoxide, and free iron [76,77]. The main role of HO-1 has been demonstrated in various diseases arising as a result of oxidative and inflammatory insults [76]. HO-1 expression is upregulated through different cell pathways (in particular the redox-dependent Nrf2 pathway) to protect against inflammation and oxidative stress [77]. HO-1 is normally present in the gastrointestinal mucosa [78] and the involvement of its expression in the regulation of intestinal barrier dysfunction was demonstrated in several in vitro and in vivo models of intestinal damage following inflammation and oxidative stress [78,79,80,81]. In our study, none of the treatments per se affected the basal levels of TNF gene expression (data not shown). Results demonstrated that TNF-α did not affect HO-1 basal gene expression levels, whereas both the Au@LCCs dispersions (1:1 and 2.6:1) induced the expression of this Nrf2-regulated gene (Figure 11), with a dose-dependent effect observed with the 2.6:1 dispersion. In agreement with our data, a similar activity was previously demonstrated by other authors in human vascular endothelial cells [82] and in human neural stem cells exposed to amyloid-beta peptide [83], since gold nanoparticles were reported to induce HO-1 expression through Nrf2 activation. Thus, it is evident that the functionalization with LCCs allows a better interaction of Au NPs with targets involved in the redox status and inflammatory signaling, very likely by improving their intracellular uptake. Furthermore, the present findings demonstrate that the optimal AuNPs:LCCs ratio needs to be established for obtaining the best anti-inflammatory and antioxidant effect.

## 4. Discussion

Monocultures based on the Caco-2 cell line are one of the main models used for in vitro nanotoxicity assessments [84]; in fact, differentiated Caco-2 cells are morphologically similar to enterocytes of the small intestine, since they polarise, form a brush border, and show functional TJ. This is of particular importance since NMs safety needs to be assessed by aligning nanotoxicology studies to the 3Rs principles (Replacement, Reduction, and Refinement of animal testing) [85]. Models employing differentiated Caco-2 cells may help to better understand the mechanisms involved in perturbations of intestinal cell functions and, in particular, in the etiology of gut inflammation, which is often associated with several pathological conditions, including inflammatory bowel diseases and metabolic syndrome.

In this work, Au, LCC, and Au@LCC nanocolloids (at the 1:1 and 2.6:1 ratio) show no toxicity on NIH/3T3 fibroblasts. On the other hand, in differentiated Caco-2 cells exposed to TNF-α, no anti-inflammatory activity was found for Au NPs and LCCs used alone. Further, TNF overexpression was dose-dependently inhibited by the pretreatment with the two Au@LCCs dispersions tested. Moreover, these Au@LCCs dispersions induced the expression of the Nrf2-regulated gene HO-1, reported to protect against inflammation and oxidative stress also at a gastrointestinal level [77,78]. Hence, also on the basis of this literature evidence, the optimal ratio AuNPs:LCCs to obtain the best anti-inflammatory and antioxidant effect was investigated and established. It resulted that the effect of the Au@LCCs 2.6:1 is higher than that of the 1:1 dispersion, very likely allowing the functionalization with LCCs and better interaction of Au NPs with targets involved in the inflammatory pathways. We remark that the selection of the optimal Au:LCCs ratio is fundamental to obtaining the best interaction between Au@LCCs and cells, and thus the wanted protective effect. Further analyses must be carried out to identify the fundamental factors governing both cell uptake and interaction mode with Au@LCCs and biomolecules. At this stage of our study, it emerged that Au-LCCs interactions promote first colloidal stability and also increase biocompatibility with respect to the bare Au NPs, so appearing LCCs are able to module the Au NP-cell interactions. In light of these preliminary findings, it is evident that Au@LCCs could have interesting clinical applications in the prevention and treatment of inflammation- and oxidative stress-related pathological conditions, especially at the intestinal level. However, further investigations are warranted to more clearly elucidate the mechanisms involved in the observed Au@LCCs protective effect, especially concerning the cell signaling pathways modulated by Au@LCCs.

## 5. Conclusions

Au@LCC nanocolloids prepared by PLAL technique showed a significant anti-inflammatory and antioxidant with respect to the bare Au NPs and LCCs. For the first time these results have been found for LCC-based nanosystems and, although preliminary, are very interesting in view of effective biomedical applications. However, this behavior is not yet fully understood. It is well known that Au interactions with LCCs (as Au charge transfer vs LCCs or chemical bonds) produce modifications in the chain electronic structure with a tendency toward a cumulenic configuration, so tuning the electronic and structural configurations of these carbon nanostructures. In the future, a deeper investigation of Au@LCCs interactions will be carried out and the optimal AuNPs:LCCs ratio should be established to obtain the best anti-inflammatory effect. Our findings can facilitate the exploitation of this innovative biomaterial for future clinical and health applications.

## Data Availability

The data presented in this study are available on request from the corresponding author.

## Abbreviations

The following abbreviations are used in this manuscript:

Au	Gold
LCC	Linear Carbon Chain
NPs	Nanoparticles
PLAL	Pulsed Laser Ablation in Liquid
STEM	Scanning Transmission Electron Microscopy
XPS	X-ray photoelectron spectroscopy
TEER	Trans-Epithelial Electrical Resistance
SPR	Surface Plasmon Resonance
