# Correction: Condorelli et al. Nano-Hybrid Au@LCCs Systems Displaying Anti-Inflammatory Activity. *Materials* 2022, *15*, 3701

**DOI:** 10.3390/ma15175986

**Published:** 2022-08-30

**Authors:** Marcello Condorelli, Antonio Speciale, Francesco Cimino, Claudia Muscarà, Enza Fazio, Luisa D’Urso, Carmelo Corsaro, Giulia Neri, Angela Maria Mezzasalma, Giuseppe Compagnini, Fortunato Neri, Antonina Saija

**Affiliations:** 1 Department of Chemical Sciences, University of Catania, V.le A. Doria 6, 95125 Catania, Italy; 2 Department of Chemical, Biological, Pharmaceutical, and Environmental Sciences, University of Messina, Viale F. Stagno D’Alcontres 31, 98166 Messina, Italy; 3 Department of Mathematical and Computational Sciences, Physical Sciences and Earth Sciences, University of Messina, Viale F. Stagno D’Alcontres 31, 98166 Messina, Italy

The authors wish to make the following correction to the published paper [1]:

## Figure/Text Correction

The original paper erroneously reports in **Figure 6** data that do not belong to the analyzed samples. Therefore, Figure 6 and the corresponding discussion must not be considered part of the work.

A correction has been made to ** Materials and Methods **, ** Au@LCC Synthesis and Characterization **, ** 2.1 **:

The last sentences now read: ** XPS spectra were acquired using a Thermo Scientific system, equipped with a monochromatic Al-Ka source (1486.6 eV), operating in a constant analyzer energy (CAE) mode with a pass energy of 20 eV for high-resolution spectra and a spot size of 400 μm. X-ray diffraction (XRD) patterns were recorded in the 2 theta range from 20° to 80° using a Bruker D8 Advance X-ray diffractometer with Cu Ka radiation (1.5406 Å). **

A correction has been made to ** Results **, ** Physico-Chemical Characterization of Au@LCC Nanocolloids **, ** 3.1 **:

**The original Figure 6 and the corresponding explanatory text have been removed and the subsequent figures have been renumbered accordingly.**

The authors apologize for any inconvenience caused and state that the scientific conclusions are unaffected. The original publication has been updated.

## References

[1] Condorelli M., Speciale A., Cimino F., Muscarà C., Fazio E., D’Urso L., Corsaro C., Neri G., Mezzasalma A.M., Compagnini G. (2022). Nano-Hybrid Au@LCCs Systems Displaying Anti-Inflammatory Activity. Materials.

